# From Storytelling to Facebook: Content Biases When Retelling or Sharing a Story

**DOI:** 10.1007/s12110-022-09423-1

**Published:** 2022-04-30

**Authors:** Alberto Acerbi

**Affiliations:** Centre for Culture and Evolution, Division of Psychology, Brunel University London, Uxbridge, UB8 3PH UK

**Keywords:** Cultural transmission, Cultural evolution, Transmission chain experiments, Cultural attraction, Content biases, Digital media

## Abstract

Cultural evolution researchers use transmission chain experiments to investigate which content is more likely to survive when transmitted from one individual to another. These experiments resemble oral storytelling, wherein individuals need to understand, memorize, and reproduce the content. However, prominent contemporary forms of cultural transmission—think an online sharing—only involve the willingness to transmit the content. Here I present two fully preregistered online experiments that explicitly investigated the differences between these two modalities of transmission. The first experiment (*N* = 1,080 participants) examined whether negative content, information eliciting disgust, and threat-related information were better transmitted than their neutral counterpart in a traditional transmission chain setup. The second experiment (*N* = 1,200 participants) used the same material, but participants were asked whether or not they would share the content in two conditions: in a large anonymous social network or with their friends, in their favorite social network. Negative content was both better transmitted in transmission chain experiments and shared more than its neutral counterpart. Threat-related information was successful in transmission chain experiments but not when sharing, and finally, information eliciting disgust was not advantaged in either. Overall, the results present a composite picture, suggesting that the interactions between the specific content and the medium of transmission are important and, possibly, that content biases are stronger when memorization and reproduction are involved in the transmission—as in oral transmission—than when they are not—as in online sharing. Negative content seems to be reliably favored in both modalities of transmission.

**Supplementary Information:**

The online version contains supplementary material available at 10.1007/s12110-022-09423-1.

Evolutionary approaches to culture are used to shed light on contemporary cultural dynamics, such as the evolution of programming languages (Valverde and Solé 2015), the prevalence of certain narrative techniques in films (Sobchuk and Tinits 2020), the diffusion of music sampling traditions (Youngblood 2019), or the cognitive underpinnings of vaccine hesitancy (Miton and Mercier 2015).

The toolbox of cultural evolution researchers includes transmission chain experiments (Mesoudi and Whiten 2008). Transmission chain experiments are a laboratory analog of the broken telephone game: in a typical setup, a first participant is presented with a piece of information, often a short story, and is asked to repeat the story to a second participant, who, in turn, will repeat it to a third, and so on, until the last member of the chain is reached. In this way, it is possible to track the transformations the story undergoes, which new details are added and, mostly, which details are lost or survive through the chain.

Transmission chain experiments often highlight the presence of cognitive biases (Stubbersfield et al. 2017) or cognitive factors of attraction (Scott-Phillips et al. 2018): certain types of information, possibly because of general evolved cognitive preferences, are more appealing or memorable and tend to be retained and transmitted with more success than others (Sperber and Hirschfeld 2004). As an illustration, when presented with a story containing both positive and negative elements, participants tended to remember and transmit preferentially negative elements, suggesting a bias toward negative information (Bebbington et al. 2017).

The same cognitive preferences that influence loss and retention in transmission chain experiments are likely to have an aggregate effect on population-level, real-life cultural dynamics. The cultural success of a maladaptive practice such as bloodletting, for example, has been linked to its cognitive attractiveness, and transmission chain experiments showed that, when presented with vignettes about bloodletting or with an alternative therapeutic practice, the bloodletting version tended to be transmitted with more success (Miton et al. 2015). Cognitive biases highlighted in transmission chain experiments have been explored in domains such as urban legends (Stubbersfield et al. 2017) or online misinformation (Acerbi 2019b). A negative bias may contribute to explain a decrease in positive emotionality in English language song lyrics over the past 50 years (Brand et al. 2019) or in Anglophone literary fiction in the past two centuries (Morin and Acerbi 2017).

However, the extension from transmission chain experiments to real-life cultural dynamics is not free from issues. If we zoom into the process of cultural transmission, the usual transmission chain setup resembles oral transmission, where individuals need to pay attention to the information they are exposed to, understand it, memorize it, and then reproduce it to other individuals with communicative intent. (A difference with respect to oral transmission is instead that, in the experiments, individuals generally are not allowed to choose whether or not to transmit a piece of information.) This contrasts with many instances of real-life cultural transmission. In the cases of music and literature mentioned above, usually individuals do not need to memorize the content they intend to transmit, and intentional modifications are often an important part of the process. Even starker is the contrast with digitally mediated transmission, a prominent contemporary example. An online sharing does not require memorization or reproduction and, in fact, it does not necessarily imply understanding, but only the willingness to transmit the content (Acerbi 2019a).

It is then critical to consider how we can generalize the results of transmission chain experiments to real-life cultural domains, or, more broadly, whether and how the details of the process of cultural transmission influence the content that is more likely to be spread. In cultural evolution research, the experiments described in Eriksson and Coultas (2014) are the first transmission chains that explicitly distinguished three separate phases of transmission: choose-to-receive (do participants want to read a story or not?), encode-and-retrieve (the standard transmission chain procedure), and finally, choose-to-transmit (do participants want to transmit the story they read or not?). The results showed that the content the experiments were focusing on—that is, content eliciting disgust—was more successful in all three phases. Stubbersfield et al. (2015) compared the performance of social information and survival information in the same three phases of transmission, showing that social information was advantaged over survival information only in the encode-and-retrieve phase, but not in the other two. Van Leeuwen et al. (2018) focused on the choose-to-transmit phase, finding limited support both for a negative bias and for an advantage of information eliciting emotions in general. Finally, Stubbersfield et al. (2018) used a modified transmission chain setup, where participants did not need to recall the story, but were invited to modify it to make it more appealing to the successive readers.

The experiments presented here aimed at explicitly comparing the same material in a standard transmission chain experiment, involving only the encode-and-retrieve phase, with a modality of transmission inspired by online sharing, involving only the choose-to-transmit phase. The experiments investigated three specific content biases that were found successful in previous research using transmission chains: negative content (Bebbington et al. 2017), threat-related information (Blaine and Boyer 2018), and information eliciting disgust (Eriksson and Coultas 2014).

A negativity bias is consistent with a broad evolutionary logic that would advantage negative information in salience and memorability, being associated with the avoidance of possible dangers (Baumeister et al. 2001). This is in agreement with previous research that found a bias toward negative sentiments in recall in transmission chain experiments (Bebbington et al. 2017), or in acceptance of information (Fessler et al. 2014), and it could influence the diffusion of information online as well (Acerbi 2019b; Bellovary et al. 2021; Melumad et al. 2021; Schöne et al. 2021). According to the same line of reasoning, it has been proposed that the information being advantaged would not generally be negative information, but a specific subset of it—that is, information concerning possible threats (Blaine and Boyer 2018). Finally, an adaptation to prevent disease can explain the salience of stimuli eliciting disgust (Curtis et al. 2004). Transmission chain experiments found that narratives with disgusting elements were better remembered and transmitted than corresponding vignettes without those elements (Eriksson and Coultas 2014). Disgust-eliciting particulars have been linked to the success of material such as urban legends (Heath et al. 2001; Stubbersfield et al. 2017) or European etiquette norms (Nichols 2002).

The first experiment reproduced an online version of the standard transmission chain setup. For each content bias, I compared the proportion of information successfully transmitted in two chains: one with a version of the story containing the attractive information (in the sense of being cognitively attractive, or more likely to be remembered and retransmitted), and one without. In the second experiment, other participants were instead asked whether they would have shared the same story (either the attractive or the neutral version) in two conditions: with their friends in their favorite social media, or anonymously in a large social media. These two conditions capture the important difference between anonymous and non-anonymous online behavior (Bernstein et al. 2011). The kinds of information shared online, and their tone, are influenced by whether they are shared anonymously or not (Correa et al. 2015). Many reasons can determine this difference, including that anonymous sharing does not affect the reputation of the individuals involved (Boyer 2018).

The results showed that negative content and threat-related information were, as predicted, favored in the standard transmission chain setup, but not, surprisingly, information eliciting disgust. Negative information was also favored when participants were asked to share it (both when sharing with friends and in the anonymous condition), but threat-related information was not. Information eliciting disgust, finally, was not favored in the sharing experiment, consistently with the transmission chain outcome.

The analyses of both experiments are conducted on data pulled together from two repetitions of the same pre-registered experiments. In the Electronic Supplementary Material (§4) I detail the reasons for this choice, and I present the results of the originals and the (exact) replications separately. Ethics approval for the study was granted by the College of Health, Medicine and Life Sciences Research Ethics Committee of Brunel University London (ref: 24117-MHR-Sep/2020–27910-2). The two experiments were fully preregistered at https://osf.io/wf7pd. All data and code to reproduce the analysis and the figures presented here can be found at https://osf.io/5yh4u/.

## Experiment 1

### Materials and Methods

For the first experiment, 1,080 participants from the UK were recruited online through *Prolific* (60% females, mean age = 36.2, SD = 12.6). Participants were pre-screened for being at least 18 years old, for reporting English as their first language, and for using a tablet or a computer desktop (not a mobile phone). Each participant was paid 0.48£, or 9.60£/hour for an estimated completion time of 3 min.

For each type of content bias (negative, disgust, threat), 120 independent chains of transmission were run, each including three participants. Three iterations are standard in transmission chain experiments, and it is considered sufficient to reveal the effect of content biases (e.g., Stubbersfield et al. 2015). Sixty chains involved the attractive content where the content bias was present, and 60 involved the content where it was not—the neutral content.

Three vignettes were used to represent the three types of content bias. Each story was a short text of five or six sentences. Attractive and neutral contents were represented by the same story, with only one detail changed. For content eliciting disgust, for example, the story involved an outbreak of an infectious disease in the fictional Saint Rika hospital in the US; it was the largest outbreak in the past 25 years, and 500 cases were identified. In the attractive version, it was reported that “the likely source of the outbreak is contact with contaminated feces in the hospital’s toilets,” whereas the neutral reported that the disease could “be transmitted when a person touches another one.” For all three stories, both the attractive and neutral variant can be found in the ESM (§1).

For each chain of transmission, the first participant read the original text and, when ready, they were asked, on a new screen, “to rewrite the story as they were retelling it to a friend.” The text generated was then provided to the second participant, and the procedure repeated by passing the text to the third and last participant. The experiment was realized with the software *Qualtrics*.

The text produced by the participants at each step of the chain was analyzed by two coders (the author and an independent coder unaware of the experimental procedure and of the predictions) for half of the chains, and by one coder (the author) for the other half. The coding consisted of determining the presence or absence of basic information from the original story. For content eliciting disgust, for example, the following were considered: (1) the name of the disease, (2) that the story took place in the US, (3) in a hospital, (4) the name of the hospital, (5) that the outbreak was the largest in X (any number of) years, (6) the correct number of years, (7) that X (any number of) cases were identified, (8) the correct number of cases, and (9) the likely source of the outbreak (or the mean of transmission, for the neutral variant). Between nine and eleven pieces of information were considered for each variant for the three content biases. Complete lists can be found at: https://osf.io/5yh4u/.

Empty texts or texts clearly not related to the task (e.g., participants writing “I could not read the story”) were excluded, and participants were replaced. For each of the three content biases, the output, consisting of the proportion of information transmitted at each step of the chain, was analyzed using generalized linear mixed models, with the position in the chain and content (attractive/neutral) as fixed effects, and each chain ID and repetition (original/replication) as random intercepts. The analysis was performed with the software R, using the *lme4* package (Bates et al. 2015). The prediction was that, for each content bias, the attractive content would be transmitted better than its neutral counterpart.

### Results

**Fig. 1:**
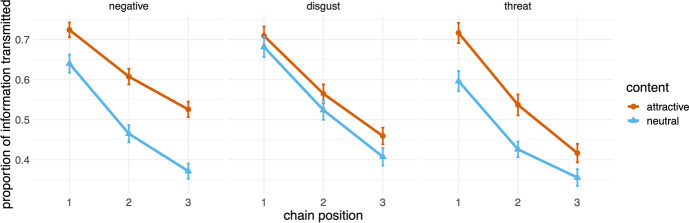
Proportion of information transmitted for the three content biases in the transmission chain setup of experiment 1 (Left: Negative content. Centre: Disgust-eliciting content; Right: Threat-related content). Points indicate the means, and error bars indicate standard errors

Overall, for experiment 1, intercoder agreement was high (94% probability of agreement, Cohen’s κ = 0.879). As expected, the proportion of content retained and transmitted decreased in all transmission chains (Fig. 1). The attractive content was better transmitted than the neutral content, confirming the predictions, for negative information (β = 0.126, *p* < 0.001) and threat-related information (β = 0.098, *p* < 0.001), but there was no difference for information eliciting disgust (β = 0.040, *p* = 0.189).

## Experiment 2

### Materials and Methods

The second experiment involved 1,200 participants from the UK, recruited online through *Prolific*, 600 participants for condition 1 (69% females, mean age = 35.82, SD = 11.4) and 600 participants for condition 2 (69% females, mean age = 34.58, SD = 11.4). Participants were pre-screened for being at least 18 years old and for reporting English as their first language (the usage of a mobile phone was allowed in this case since participants did not have to produce written text). Each participant was paid 0.73£, or 8.76£/hour for an estimated completion time of 5 min.

Experiment 2 used exactly the same material used as seeds for the transmission chains of experiment 1. In this case, however, the three texts, one for each content bias, were presented to each participant, in random order. For each content bias, again randomly, either the attractive or the neutral version was presented. In condition 1 (“anonymous”), for each text, participants were asked if they would share the story “anonymously, in a large social network, such as Reddit.” In condition 2 (“sharing with friends”), the participant was asked if they would share the story “with your friends in your favorite social media.” The experiment was also realized with the software *Qualtrics*.

The output for each content bias, consisting of the decision of sharing or not, was analyzed using generalized linear mixed models (binomial) with content (attractive/neutral) as fixed effect and order of presentation and repetition (original/replication) as random intercepts. As in experiment 1, the analysis was performed with the software R, using the *lme4* package (Bates et al. 2015). In this case, there was no specific prediction, but the research question was whether in each condition (anonymous/sharing with friends), and for each content bias, the attractive content was shared more than the neutral content.

### Results

**Fig. 2:**
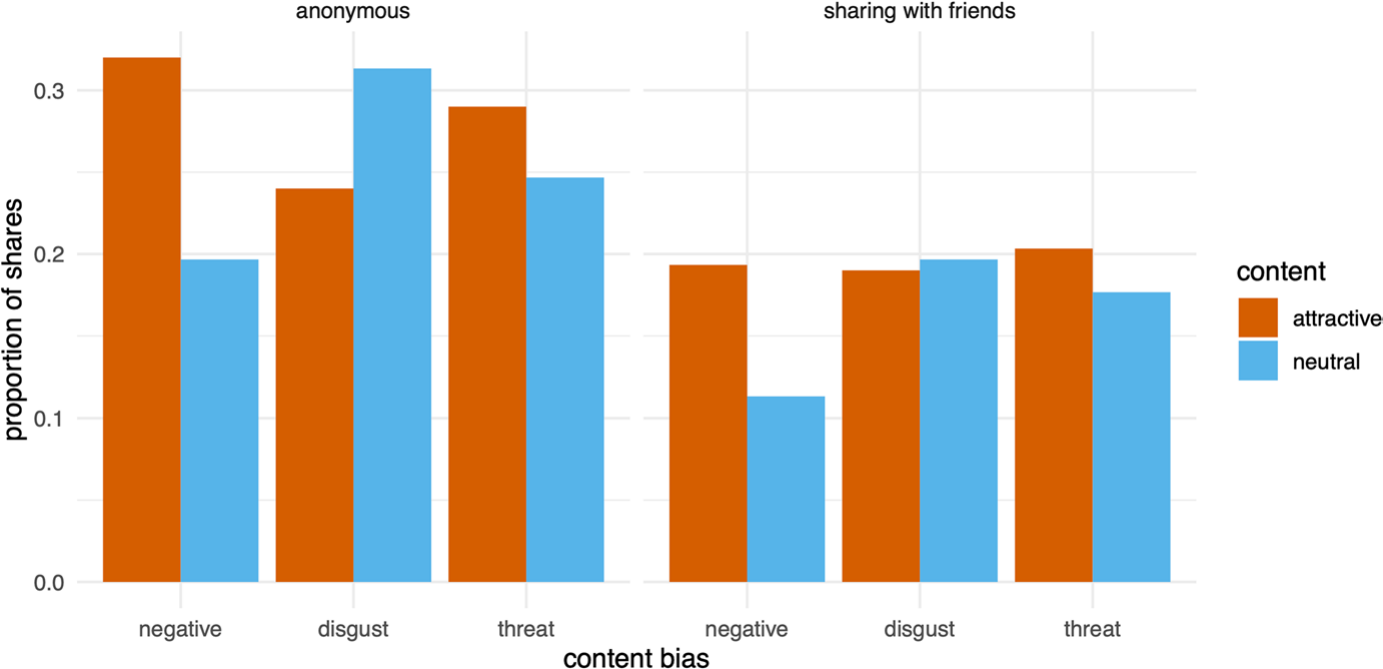
Proportion of shares for the two conditions of experiment 2, for the three content biases (Left: “anonymous sharing” condition; Right: “sharing with friends” condition)

In experiment 2 (Fig. 2), the attractive content was shared more than the neutral counterpart for only one content bias—negative content—both in the anonymous (β = 0.644, *p* < 0.001) and in the “sharing with friends” (β = 0.607, *p* = 0.010) condition.

The difference was also significant for information eliciting disgust in the anonymous condition, but in the unexpected direction, with neutral content shared more than attractive content (β =  − 0.370, *p* = 0.046), while no significant difference was found in the “sharing with friends” condition (β =  − 0.061, *p* = 0.769). Finally, no effect was found for threat-related information either in the anonymous (β = 0.231, *p* = 0.213) or “sharing with friends” (β = 0.165, *p* = 0.428) condition.

## Discussion

The results provided a somewhat composite picture. The outcomes of experiment 1 (resembling oral transmission) and experiment 2 (resembling online sharing) were consistent for negative information and disgust-eliciting information, with the former being advantaged, and the latter not, in both experiments. Threat-related information, instead, was advantaged in transmission chain experiments, but not in the sharing condition.

The clearest result is the advantage of negative information both in standard transmission chains and in the sharing experiments, consistent with previous literature showing a ubiquitous negative bias (discussed above). On the other side, the present experiments compared a negative narrative with a neutral one, without explicitly considering a positive condition. Other transmission chain experiments indeed found an advantage for emotional content in general (Stubbersfield et al. 2017), and, as above, emotional content, independent of the direction (positive or negative), has been found to favor online diffusion of content (Berger and Milkman 2012; Brady et al. 2017).

Surprisingly, content eliciting disgust was not transmitted or shared differently from its neutral counterpart. In fact, in the anonymous sharing condition of experiment 2, the attractive content was transmitted *less* than the corresponding neutral content. This result suggests that fine-grained details of the transmission process may be important for the resulting cultural dynamic. For example, we may be attracted by particular content, and perhaps remember it better, but we may not be willing to share it (an obvious example is sex-related information: Berriche and Altay 2020). Similarly, subtle cues in the experimental procedure may or may not favor the repetition of sensitive material, such as the perception of anonymity, whether participants think that their text will be passed to others, and so on. Another option is that both versions of the story involved a scenario presenting a risk of exposure to pathogens so they were both attractive, not because of eliciting disgust but because of the activation of our behavioral immune system. Still, even according to this interpretation, stimuli eliciting disgust should be *particularly* relevant for the behavioral immune system (Curtis et al. 2011) and possibly resulting in its being advantaged.

Threat-related content was the only content bias for which a difference between the transmission chain experiments and the sharing experiments (in both conditions) was found, with the attractive content being advantaged in the transmission chains but not in the sharing scenario.

The overall contrast between transmitting and sharing, with respect to content biases, is not straightforward. A possible suggestion, worth exploring in future studies, is that the effect of content biases is stronger when retelling a story than when sharing it. The attractive content was favored in two cases out of three in the transmission chain experiments (negative and threat-related content) and two out of six possible combinations of bias/condition in the sharing experiments (negative content on both anonymous and sharing conditions). This difference is clearer when considering the repetitions of the experiments (original and replication) separately (see ESM §4). In this case, attractive content was advantaged four times out of six in the transmission chain experiments and only in two out of twelve possible cases in the sharing experiments. This difference would be consistent with the idea that cognitive factors related to memorization and reproduction influence the content biases so that they would be stronger when the medium of transmission requires these phases. More counterintuitively, it would suggest that online sharing may be *less* content-biased than oral transmission. This may even contribute, together with other reasons, to explain the “paradox” (Altay et al. 2020) in which fake news, despite being cognitively attractive (Acerbi 2019b), has a limited diffusion online (e.g., Allen et al. 2020; Guess et al. 2019).

Regarding the difference between anonymous and non-anonymous sharing, the effect of the attractive content was not evident. In the case of negative content, the only one in which attractive content was favored, it was favored in both conditions. The main difference was that sharing was, in general, less common in the “sharing with friends” than in the “anonymous sharing” condition. In the former, the total sharing was around two-thirds of the latter (322 vs. 482). It is also interesting to note that the total amount of sharing was less than expected; since, overall, the possible occasions to share were 1,800 for each condition, participants shared less than 20% and 30% of the times, respectively. This may be explained by the fact that stories were possibly interesting but lacked personal relevance for participants, and this consideration would be even more important in the “sharing with friends” condition, where reputational concerns could have been involved (Altay et al. 2020).

Some of these results may be explained by the limitations of the current study. First, participants were not actually sharing content in social media (and, as a consequence, they were also neither anonymous nor openly shared among friends), they were simply asked if they *would* share that content. Even though self-reported sharing intentions have been shown to be relatively good indicators of real online sharing behavior (Mosleh et al. 2020), subtle differences could change the outcomes presented here. Second, small sharing advantages for a type of content could be amplified in real social media by actual sharing. Individuals would then encounter more of the attractive versions, whereas here the attractive and neutral versions were presented at the same rate. A possible extension that addresses these shortcomings may be represented by digital field experiments, such as having a bot post attractive/neutral material (of various contents) in real social media and see how the posts are shared.

In the analysis of experiment 1, consistent with transmission chain studies in cultural evolution literature (e.g., Mesoudi et al. 2006; Stubbersfield et al. 2015), I chose to consider the recall and transmission of information of the whole story presented to participants. The assumption is that the presence of a piece of attractive information would make the whole story more attractive. An alternative analytical strategy would be to consider the performance on only the key pieces of information that distinguish the two versions of the stories. For example, for the content eliciting disgust they would be the reference to feces/toilets as the source of the outbreak and the reference to touching another person as the mean of transmission, for the attractive and the neutral version, respectively. I report these results in the ESM (§3). They confirm the main outcome of negative information being better transmitted than its neutral counterpart, but the effect was reversed for the other two contents, with an advantage for the content eliciting disgust, consistent with previous literature, but no effect for threat-related information. Few studies have explicitly considered how the presence of attractive information influences the performance of the associated (not attractive by itself) information (e.g., Mermelstein et al. 2021). Along with determining how different analytical strategies related to this difference can influence the interpretation of the results, these are important questions for future transmission chain studies.

Another aspect concerns possible differences between the stories presented to participants. Stories used in transmission chain experiments labelled as, say, “negative” are different from each other, making comparisons difficult. Even more, comparing the effect of different content biases, as in the experiments reported here, risks obscuring effects specific to the stories. Although stimulus checks ensured that the attractive versions were different from the neutral ones (see ESM §2), the differences between content biases could be due to the differences in how much more attractive one story was with respect to its counterpart. For example, the difference in rating between the attractive version and the neutral version of the negative story was stronger than for the two other content biases, possibly contributing to the overall result of negative information being advantaged in both experiments. As for the previous point, systematic transmission chain studies comparing both different narratives within the same content bias and different content biases could be useful to make progress in this direction.

More generally, these experiments point to the importance of keeping into account the details of the transmission process when considering which traits will be favored by cultural evolution. If content biases such as the ones studied here can make a cultural trait appealing and memorable, the decision of transmitting it can be detached from these qualities. The types of news that people tend to read most are in general different from the types of news they share most (Bright 2016). Sharing can be motivated by various interests, including managing one’s reputation (Altay et al. 2020) and signalling group membership (Osmundsen et al. 2021), that are not necessarily aligned with the intrinsic attractiveness of a piece of information. Different contents can have different advantages depending on different intentions and modalities of transmission. Ultimately, cultural evolution has mostly focused on consumers of cultural traits as a determinant of cultural success—what they want to copy, from whom—while less attention has been given to the motivation of the transmitters or producers (André et al. 2020). This may be a fruitful direction for future studies.

## Supplementary Information

Below is the link to the electronic supplementary material.

Supplementary file1 (PDF 253 KB)

## Data Availability

All anonymized data and material to reproduce the results described in the manuscript are available in an OSF repository online at: https://osf.io/5yh4u/ The experiments were fully preregistered. Preregistration available at https://osf.io/wf7pd

## Code Availability

The code to analyze the data is available in an OSF repository online at: https://osf.io/5yh4u/
