# Supplementary material for: From Storytelling to Facebook: Content Biases When Retelling or Sharing a Story
Source: Hum Nat. 2022 Apr 30;33(2):132–44. doi: 10.1007/s12110-022-09423-1 (PMC9250454; doi:10.1007/s12110-022-09423-1)
Supplement: Supplementary file 1 — Supplementary file1 (PDF 253 KB) [file 12110_2022_9423_MOESM1_ESM.pdf]

Supplementary Information for: From storytelling to Facebook. Content biases when retelling or sharing a story

Alberto Acerbi

Human Nature 33(2), June 2022, https://doi.org/10.1007/s12110-022-09423-1

1. Full texts of stories

Negative

Attractive: A woman from Oregon hit the $8 million jackpot at a Lucky Eagle Casino slot machine. Veronica Castillo took her mother to the casino in Rochester, Washington. She put $100 in a machine and hit the jackpot. The casino staff came over and told her the machine malfunctioned. The woman attempted a legal battle, but she could not claim her money.

Neutral: A woman from Oregon hit the $8 million jackpot at a Lucky Eagle Casino slot machine. Veronica Castillo took her mother to the casino in Rochester, Washington. She put $100 in a machine and hit the jackpot.The casino staff came over and congratulated her and her mother. It was the highest win from the opening of the casino.

Eliciting disgust

Attractive: A major outbreak of salmonella has been reported in the US. The outbreak is the largest in the last 25 years. The outbreak had its origin in Saint Rika's hospital. More than 500 cases were identified in the hospital only. The likely source of the outbreak is contact with contaminated faeces in the hospital's toilets.

Neutral: A major outbreak of chickenpox has been reported in the US. The outbreak is the largest in the last 25 years. The outbreak had its origin in Saint Rika's hospital. More than 500 cases were

identified in the hospital only. Chickenpox can be transmitted when a person touches another one.

Threat-related

Attractive: The FBI reported increases in the violent crime rate between 2017 and 2019. Violent crime includes offences such as rape, robbery and assault. Most of the crimes that are reported to police are not solved. This is based on an FBI measure known as the "clearance rate." In 2018, police nationwide cleared less than half of violent crimes that were reported to them.

Neutral: The FBI reported increases in corporate bribery between 2017 and 2019. Corporate bribery means giving a reward to influence someone's behaviour. Most of the bribery cases that are reported are not solved. This is based on an FBI measure known as the "clearance rate." In 2018, police nationwide cleared less than half of bribery cases that were reported to them.

2. Stimuli checks

To assess if there was a reliable difference between attractive and neutral stories, I tested whether attractive stories were indeed rated as more negative, electing disgust, or concerning threats than their neutral counterparts. A total of 180 participants from UK were recruited online through Prolific. Participants were pre-screened for being more than 18 years old and for reporting English as their first language. Each participant was paid 0.50£, or 15.00£/hour for an estimated completion time of 2 minutes.

Three texts, one for each content bias, were presented to each participant, in random order. For each content bias, again randomly, either the attractive or the neutral version was presented. After reading the story, the participant were asked, depending on the context bias, if they agreed with the statement "The story is negative," or "The story elicits disgust," or "The story concerns possible threats." Participants could answer using a 5-Point Likert Scale, with values (1) Strongly agree; (2) Agree; (3) Neutral; (4) Disagree; (5) Strongly disagree. The procedure, as for the main experiments, was realised with the software Qualtrics.

While to a variable degree among the three content biases (see Figure 1), in all three cases the ratings of the attractive version were significantly different, according to a Mann-Whitney U test, from the ratings of the neutral version: negative content ($n_1 = n_2 = 90, U = 380, p < 0.001$), information eliciting disgust ($n_1 = n_2 = 90, U = 906, p < 0.001$), and threat-related information ($n_1 = n_2 = 90, U = 2108, p < 0.001$).

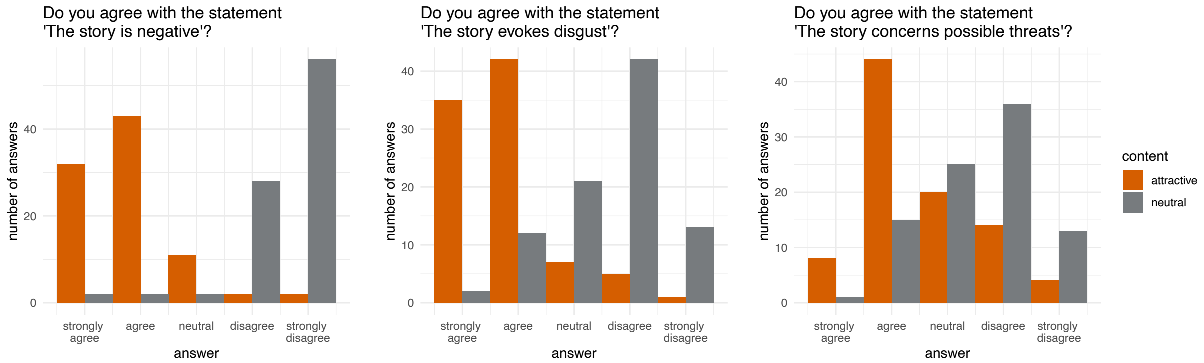

Figure 1: Rating on attractive and neutral stories for the three content biases.

3. Key information analysis

The analysis reported in the main manuscript for Experiment 1 focused on determining the presence or the absence of information from the full story presented to participants. An alternative strategy could be tracking only the key information, i.e. the pieces of information that distinguish the attractive and neutral material. The key information was defined as such:

Negative

Attractive: References to (i) the machine malfunctioning, (ii) that there was a legal battle (iii) that was lost

Neutral: References to (i) the fact that the staff congratulated (ii) as it was the highest win

Eliciting disgust

Attractive: References to (i) faeces/toilets as the source of the outbreak

Neutral: References to (i) touching another person as the mean of transmission

Threat-related

Attractive: References to (i) violent crimes, (ii) such as rape, robbery and assault

Neutral: References to (i) corporate bribery, (ii) meaning giving a reward to influence someone's behaviour

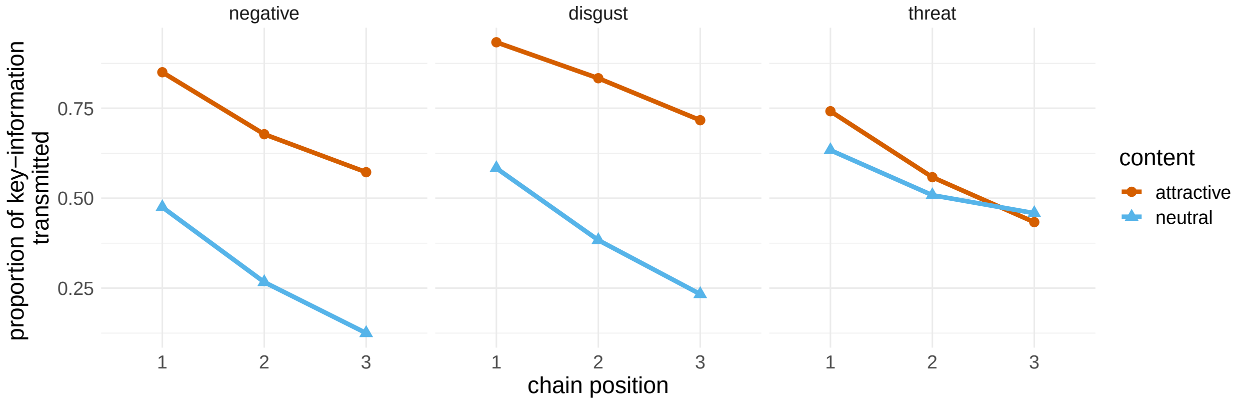

Figure 2: Proportion of key information transmitted for the three content biases in the transmission chain set-up of Experiment 1.

From a visual inspection (see Figure 2), the output is consistent with the strong effect of negative content, but in this case it seems that there is also a strong effect of the key information on disgust (faeces in the hospital toilets versus a person touching another one) and less effect for the threat-related key information.

4. Full results of original experiments and replications

After running the original experiments, a concern was that the story related to information eliciting disgust, involving an outbreak of an infective disease (see main manuscript), could have evoked associations with the COVID-19 pandemic in the participants, possibly influencing the results. Therefore, I tested two alternative vignettes, inspired by two of the stories used in Eriksson & Coultas (2014). In both cases, however, the transmission chain experiments did not show that the version with information eliciting disgust was more successful than the alternative (data not shown). Notice this can not be interpreted as a failed replication of Eriksson & Coultas (2014), as I shortened the stories (to make their length comparable with my material); because my version, differently from Eriksson & Coultas (2014), was implemented online; and, finally, because I coded the texts differently. In any case, as I become wary of my own results, I decided to replicate my own experiments. As detailed in the manuscript, for the main analysis I pulled together the data of the original experiments and of the replications. Here I report separately the results.

Original experiment

Experiment 1

For the first experiment, 540 participants (57% females, $M_{age} = 36.16, SD = 12.8$) were recruited. The methods are as detailed in the main manuscript, with the difference that 60 independent chains of transmission were run for each content bias (30 with the attractive content and 30 with the neutral content), and that the analysis did not include repetition (original/replication) as random effect.

In the original experiment, confirming the predictions, the proportion of content retained remained higher for the attractive content for all three content biases (see Figure 3): negative information ($\beta = 0.102, SE = 0.034, t(58) = 3.010, p = 0.004$), information eliciting disgust ($\beta = 0.081, SE = 0.039, t(58) = 2.065, p = 0.043$), and threat-related information ($\beta = 0.145, SE = 0.040, t(58) = 3.607, p < 0.001$).

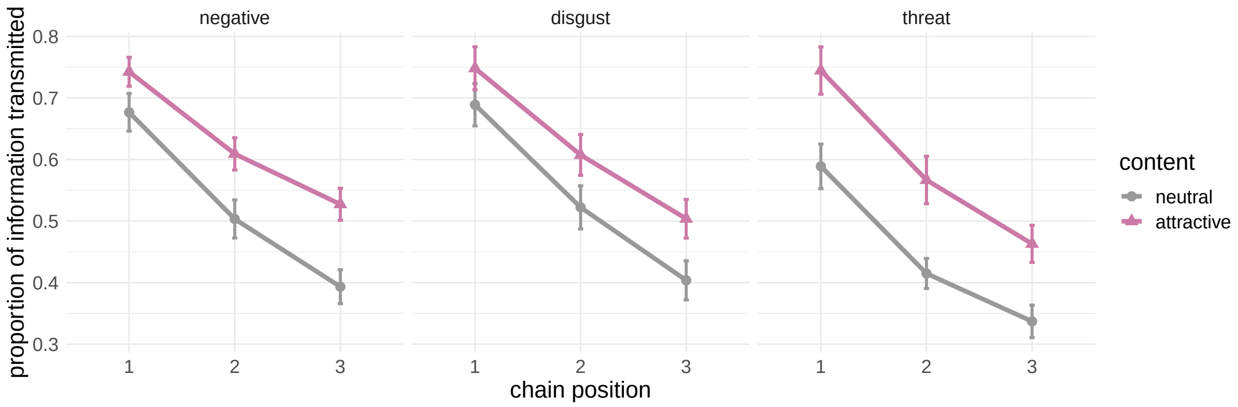

Figure 3: Proportion of information transmitted for the three content biases in the transmission chain set-up of Experiment 1 (original experiment). Points indicate the means, and error bars indicate standard errors.

Experiment 2

The second experiment involved 600 participants: 300 participants for condition 1 (76% females, $M_{age} = 34.75, SD = 11.3$), and 300 participants for condition 2 (69% females, $M_{age} = 34.15, SD = 11.1$). The methods are as detailed in the main manuscript, with the difference that the analysis did not include repetition (original/replication) as random effect.

The attractive content was shared more than the neutral counterpart in only one out of six possible combinations condition/content-bias, i.e. negative content in the anonymous sharing condition, The full results are as below (see Figure 4). Anonymous sharing condition: negative information ($\beta = 0.886, SE = 0.280, z(296) = 3.167, p = 0.002$), information eliciting disgust ($\beta = -0.270, SE = 0.245, z(296) = -1.010, p = 0.313$), and threat-related information ($\beta = 0.378, SE = 0.270, z(296) = 1.402, p = 0.161$). "Sharing with friends" condition: negative information ($\beta = 0.180, SE = 0.341, z(296) = 0.529, p = 0.597$), information eliciting disgust ($\beta = -0.197, SE = 0.291, z(296) = -0.677, p = 0.498$), and threat-related information ($\beta = 0.077, SE = 0.305, z(296) = 0.252, p = 0.801$).

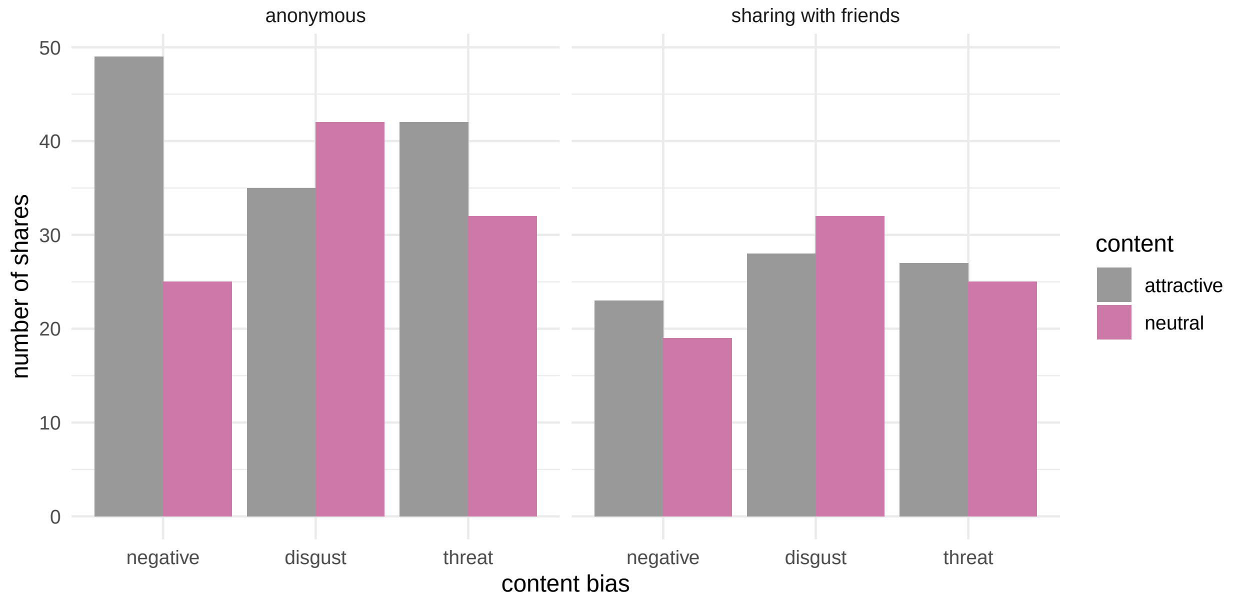

Figure 4: Number of shares for the two conditions of Experiment 2, for the three content biases (original experiment).

Replication

Experiment 1

For replication of the first experiment, 540 participants (63% females, $M_{age} = 36.47, SD = 12.4$) were recruited. As above, the methods are as detailed in the main manuscript, with the difference that 60 independent chains of transmission were run for each content bias, and that the analysis did not include repetition (original/replication) as random effect.

In the replication (see Figure 5), the proportion of content retained remained higher for the attractive content in the case of negative information ($\beta = 0.152, SE = 0.035, t(58) = 4.341, p < 0.001$), but there was no difference for information eliciting disgust ($\beta = -0.001, SE = 0.029, t(58) = -0.034, p = 0.973$) and threat-related information ($\beta = 0.051, SE = 0.031, t(58) = 1.303, p = 0.198$).

Experiment 2

The second experiment involved 600 participants (300 participants for condition 1 (62% females, $M_{age} = 36.88, SD = 11.5$), and 300 participants for condition 2 (70% females, $M_{age} = 35, SD = 11.6$). As above, the methods are as detailed in the main manuscript, with the difference that the analysis

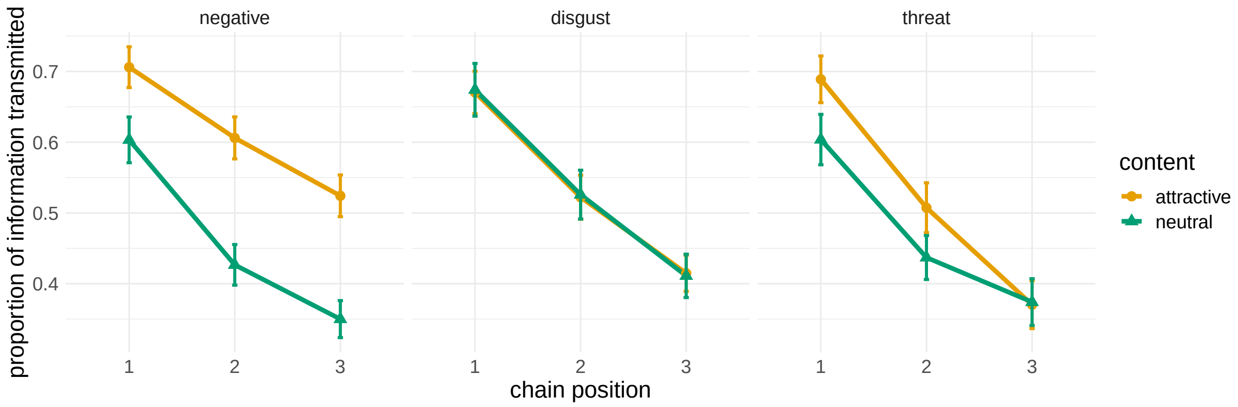

Figure 5: Proportion of information transmitted for the three content biases in the transmission chain set-up of Experiment 1 (replication). Points indicate the means, and error bars indicate standard errors.

did not include repetition (original/replication) as random effect.

In the replication, the attractive content was shared more only in the negative information/sharing with friends case (see Figure 6). The full outputs of the anonymous sharing condition were: negative information ($\beta = 0.424, SE = 0.263, z(296) = 1.615, p = 0.106$), information eliciting disgust ($\beta = -0.452, SE = 0.258, z(296) = -1.750, p = 0.08$), and threat-related information ($\beta = 0.105, SE = 0.256, z(296) = 0.410, p = 0.682$). In the "sharing with friends" condition: negative information ($\beta = 0.992, SE = 0.334, z(296) = 2.968, p = 0.003$), information eliciting disgust ($\beta = 0.117, SE = 0.299, z(296) = 0.393, p = 0.694$), and threat-related information ($\beta = 0.245, SE = 0.286, z(296) = 0.854, p = 0.393$).

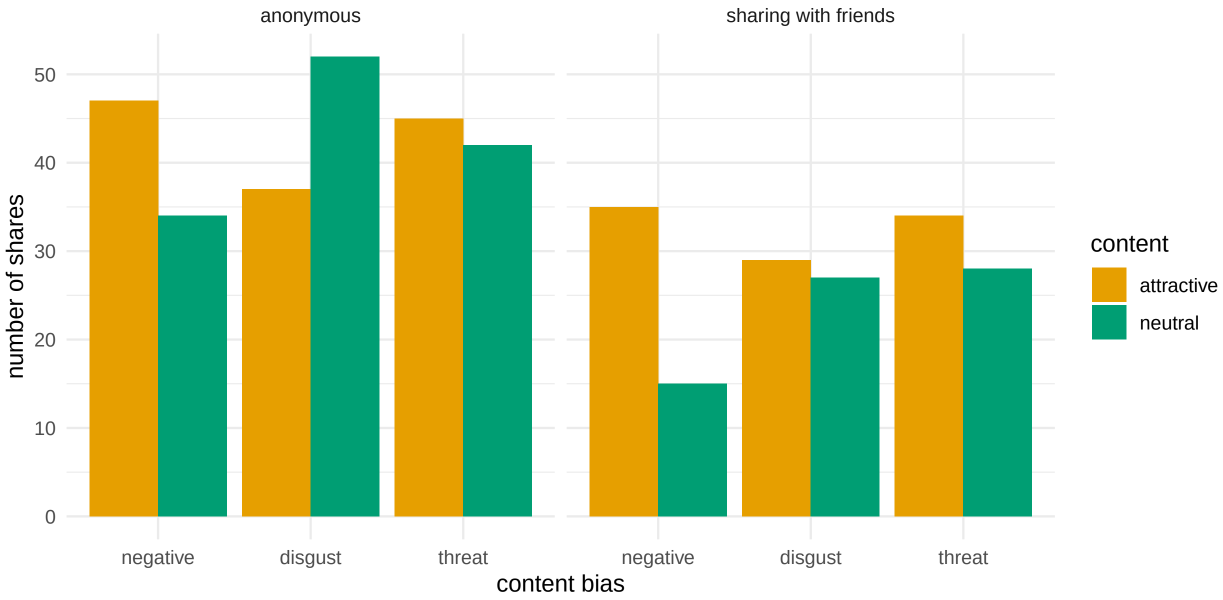

Figure 6: Number of shares for the two conditions of Experiment 2, for the three content biases (replication).

References

Eriksson, K., & Coultas, J. C. (2014). Corpses, Maggots, Poodles and Rats: Emotional Selection Operating in Three Phases of Cultural Transmission of Urban Legends. *Journal of Cognition and Culture*, *14*(1-2), 1–26. https://doi.org/10.1163/15685373-12342107
